# Inducers, Attractors and Modulators of CD4^+^ Treg Cells in Non-Small-Cell Lung Cancer

**DOI:** 10.3389/fimmu.2020.00676

**Published:** 2020-04-28

**Authors:** Mengxiao Xie, Jia Wei, Jian Xu

**Affiliations:** ^1^Department of Laboratory Medicine, The First Affiliated Hospital of Nanjing Medical University, Nanjing, China; ^2^National Key Clinical Department of Laboratory Medicine, Nanjing, China

**Keywords:** non-small cell-lung cancer, tumor microenvironment, CD4^+^ Tregs, differentiation, proliferation, chemotaxis, phenotype

## Abstract

Lung cancer is the leading cause of cancer-associated deaths worldwide, with non-small cell-lung cancer (NSCLC) accounting for approximately 80% of cases. Immune escape has been demonstrated to play a key role in the initiation and progression of NSCLC, although the underlying mechanisms are diverse and their puzzling nature is far from being understood. As a critical participant in immune escape, the CD4^+^ T cell subset of regulatory T (Treg) cells, with their immunosuppressive functions, has been implicated in the occurrence of many types of cancers. Additionally, therapies based on Treg blockade have benefited a portion of cancer patients, including those with NSCLC. Accumulating literature has noted high Treg infiltration in NSCLC tumor tissues, bone marrow, lymph nodes and/or blood; moreover, the tumor milieu is involved in regulating the proliferation, differentiation, recruitment and suppressive functions of Treg cells. Multifarious mechanisms by which CD4^+^ Treg cells are generated, attracted and modulated in the NSCLC milieu will be discussed in this review.

## Introduction

Lung cancer is continuously evaluated and is considered the leading cause of cancer-related mortality worldwide, with a dismal 5-year survival rate of approximately 19% in the United States (1, 2). According to histopathological features, lung cancer can be further classified into small-cell lung cancer (SCLC), which accounts for approximately 10% ∼ 15% of lung cancer cases, and non-small-cell lung cancer (NSCLC), comprising the remaining 85% ∼ 90% (3, 4). NSCLC has three histological subtypes: squamous cell carcinoma, adenocarcinoma, and large cell carcinoma (5). Although genetic susceptibility and environmental hazards (i.e., cigarette smoke) that trigger chronic pulmonary inflammation have been confirmed as high-risk factors for NSCLC (6); their precise regulatory mechanisms are still puzzling.

Cancer immune escape, involving (a) loss of antigenicity, (b) loss of immunogenicity, and (c) the presence of a complicated immunosuppressive tumor microenvironment, is a major mechanism antagonizing antitumor immune responses (7). Published literature has emphasized that tumor milieu-induced activation of immunosuppressive cells enhances cancer occurrence, growth, invasion, and metastasis by diminishing tumor-killing immune responses (8–11). More importantly, therapeutics targeting the contributing factors in the tumor microenvironment have achieved an unexpected breakthrough in some patients, including those suffering from NSCLC (12–14), implying the non-redundant role of immunosuppression in modulating tumor biology.

CD4^+^ regulatory T (Treg) cells, which express the X chromosome-linked, linage specific transcription factor Foxp3, are potent immunosuppressive cells and can serve as brakes during immune responses. Numerous studies on autoimmune diseases have emphasized the protective role of Treg cells in remitting inflammation and the requirement for Treg supplementation therapy in these inflammatory diseases (15). However, Treg cells play an opposite role in cancer immunity as Treg cells recruited in tumor tissues become accomplices that help cancer cells escape from immunological surveillance. Heterogenetic Tregs with high frequencies in tumor tissues, bone marrow, lymph nodes or peripheral blood from NSCLC patients are considered predictors of disease outcome (16–18). Further, accumulating evidence suggests that cytokines or other agents derived from NSCLC tissues, as well as phenotype modulators, might be the key factors leading to the differentiation, trafficking and immunosuppressive effects of Tregs. In the following sections of this review, we will discuss the underlying mechanisms in detail.

## Generation or Expansion of Treg Cells

CD4^+^ Treg cells are currently classified into two main subtypes, natural Tregs (nTregs) and inducible Tregs (iTregs), according to their origins, determinant markers and distributions (19). Despite the existence of delicate differences, both subtypes express the linage specific transcription factor Foxp3 and can exert immunosuppressive effects. Considering that conventional T cells undergo reprogramming, proliferation and differentiation in the NSCLC milieu, it is worthwhile summarizing the mechanisms of Treg cell generation induced by factors in NSCLC microenvironment.

### TCR Signaling

Recognition of antigen peptide-MHC complexes by T cell receptors (TCRs) is the first step in T cell proliferation and differentiation. An updated published work revealed that the TCR repertoires of intratumoral Tregs from patients with metastatic melanoma, gastrointestinal, and ovarian cancer have a unique TCR repertoire different from that of other intratumoral CD4^+^ T cells (20). These results emphasize the possibility that *ex vivo* generation or expansion of NSCLC-infiltrating Tregs, like other T cell subsets, also requires distinct TCR signaling in response to neoantigens, which determines their heterogeneity. This section will be discussed in the following part.

### Coinhibitory Ligands and Receptors

Following TCR stimulation, T cells undergo further proliferation and lineage fate determination subsequent to CD28-CD80/CD86 costimulatory interaction (21). Additionally, coinhibitory crosslinking, including cytotoxic T lymphocyte associated antigen-4 (CTLA-4)-CD80/86 and programmed cell death protein-1 (PD-1)-programmed death-ligand-1 (PD-L1) binding, both of which serve as brakes in the process for T cell activation, can occur.

**FIGURE 1:**
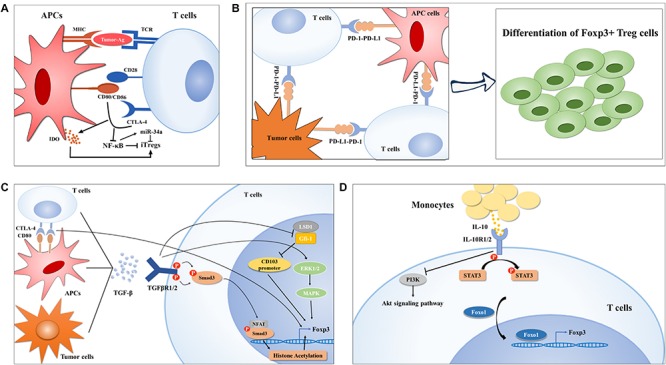
Treg cell generation in lung cancer. **(A)** *Ex vivo* generation of Tregs is modulated by the first and second signaling of T cell activation in lung cancer. In brief, neoantigens determines the TCR repertoire of Tregs (left) and CTLA-4-CD80/CD86 crosslink downregulates NF-κB activity, which was reported to inhibit Foxp3 expression by upregulating miR-34a, finally promoting Treg cell polarization. **(B-C)** APC- or tumor cell-derived PD-L1 or TGF-β can also induce Treg cell generation by interaction their corresponding receptors, respectively, on TILs via diverse mechanisms. On the one hand, TGF-β induces CTLA-4 expression on TILs, on the other hand, TGF-mediated activation of Smad and ERK1/2 can enhance Foxp3 expression in Treg cells. Moreover, TGF-β inhibits LSD1-Gfi-1 axis *via* an unknown mechanism to enhance immunosuppressive CD103^+^ Treg differentiation. **(D)** IL-10 induced Foxo1 translocation into nucleus facilities its occupation in Foxp3 promoter upon STAT3 activation and PI3K-Akt inactivation.

CTLA-4, a CD28 family receptor, is not expressed by resting T cells but can be induced by *de novo* transcription and accumulates on membranes upon T cell stimulation (22). On the one hand, CTLA-4 induced by activated T cells can compete with CD28 to interact with CD80/86 with high affinity, causing T cell anergy (23); on the other hand, it has a positive effect on iTreg cell differentiation (24). Although the current mechanisms by which CTLA-4 promotes Treg generation *in vitro* remain unelucidated, this activity could be ascribed to an emulative CTLA-4 mediated reduction in CD28-CD80/86-interaction-induced NF-κB activity, which is specially required for iTreg, but not nTreg differentiation, potentially in an miR-34a-dependent manner (25–27). Alternatively, Treg generation can be achieved *via* indoleamine 2,3-dioxygenase (IDO) production by dendritic cells (DCs) upon CTLA-4-CD80/86 interaction, which favors *in vitro* differentiation of iTregs (28–30). Emerging evidence has indicated that CTLA-4 expression level is markedly elevated in tumor-infiltrating T cells of NSCLC patients (31), which might contribute to their conversion into iTreg cells (Figure 1A). So far, two CTLA-4 monoclonal antibodies, namely ipilimumab and tremelimumab, have been developed to enhance antitumor immune responses by recovering T cell activation status (32, 33). Ipilimumab has been evaluated in advanced NSCLC in combination with chemotherapy in a Phase II study and the results showed that phased ipilimumab plus chemotherapy significantly improved progression-free survival (PFS) compared with chemotherapy alone (34). Notably, anti-CTLA-4 therapy has shown a promising outcome for decreasing Treg cell numbers, which has been mentioned and suggested for NSCLC treatment (35–37); however, the definite effect of CTLA-4-based therapies on Treg cell numbers needs further investigation.

PD-1, also called CD279, is an immune checkpoint receptor that is a CD28 family receptor and is expressed on diverse types of immune cells including Tregs (38–41). PD-L1, also termed CD274 or B7-H1, is a transmembrane protein that transmits an inhibitory signal promoting T cells to undergo apoptosis and anergy by binding to its receptor—PD-1 (42–44). Numerous studies in human NSCLC patients or a mouse model of EGFR-driven adenocarcinomas have implicated hyperactivation of the PD-1-PD-L1 axis in tumor immune escape and malignant progression (45–47), and manipulation of Treg generation driven by this axis constitutes one of the most predominant mechanisms of NSCLC occurrence (Figure 1B). Using TCR transgenic CD4^+^ OT-II T cells, Wang et al. (48) found that the conversion of OT-II T cells into iTreg cells was notably diminished after PD-L1 blockade *iv vivo*, and that the loss of PD-L1 in DCs failed to induce iTreg differentiation *ex vivo*, preliminarily suggesting a PD-L1-dependent mechanism for Treg cell generation. Chen et al. (49) showed that conventional CD4^+^ T cells with PD-1 knockout displayed a strikingly diminished tendency toward differentiation into iTreg cells, arguing that the PD-1-PD-L1 pathway, in addition to limiting conventional T cell activation and proliferation, can also provoke peripheral tolerance by supporting iTreg generation. Reportedly, genomic alterations including gene amplification and translocation at chromosome 9p24.1 (50) and genomic structural alteration in the PD-L1 3′-untranslated region (3′-UTR) (51), several epigenetic modifiers, comprising the histone acetylation modifier bromodomain and extra-terminal (BET) proteins (52, 53) and mixed-lineage leukemia 1 (MLL1) with histone methyltransferase activities (54), as well as oncogenic signaling pathways, involving JAK-STAT (55), NF-κB (56), PI3K-Akt-mTOR (57), MAPKs (58, 59), and Hippo (60), have been implicated in PD-L1 expression at different levels. Further investigations are needed to disclose the precise mechanisms underlying the hyper-induction of infiltrating Treg cells in NSCLC driven by PD-1-PD-L1 activation.

### TGF-β Cascade

Transforming growth factor beta (TGF-β) is a polypeptide cytokine of the transforming growth factor superfamily that includes three different isoforms (TGF-β1, 2, and 3) and regulates multiple biological processes involved in embryonic development, stem cell differentiation, immune regulation, wound healing, and inflammation (61–63). This pathway is initiated by TGF-β signaling through the cell-surface receptors, TGF-βR1 and TGF-βR2, which are dual specificity kinases and intracellular signal transducer proteins. Upon activation of these receptors, Smad proteins undergo phosphorylation by TGF-βR1 kinase at the two carboxy-terminal serine residues and then translocate into the nucleus to modulate target gene expression (64). In addition, non-redundant pathways through which TGF-β can also activate MAPK, Rho-like GTPase and PI3K pathways independent of Smad have been recorded (65).

Both *in vivo* and *ex vivo* investigations suggested that TGF-β signaling is required for the induction of Foxp3 in peripheral CD4^+^ T cells through different mechanisms (66–68). For instance, Smad3 can induce Foxp3 expression by binding the conserved non-coding sequence 1 (CNS1) region of Foxp3 enhancer or facilitating binding of the transcription factor nuclear factor of activated T cells (NFAT) to Foxp3 enhancer, further triggering histone acetylation at this locus (69, 70). Zheng and colleagues (24) revealed that TGF-β can accelerate the expression of CTLA-4, whose binding to CD80 shortly after T cell activation enables Foxp3 induction in conventional CD4^+^ cells and to endows them with suppressive activity, implying that TGF-β participates in the coinhibitory crosslinking between CTLA-4 and CD80. In addition, Lu and colleagues (71) documented that TGF-β boosts iTreg cell differentiation through Smad2/3 and MAPK-ERK1/2 signaling, suggesting the involvement of non-redundant Smad and non-Smad signaling in the development of Treg cells. Moreover, in the subset of CD103^+^ iTreg cells which were defined as tumor-infiltrating Tregs (72), TGF-β reportedly inhibited the expression of growth factor independent 1 (Gfi-1), a transcriptional repressor that can form a repressive complex with lysine-specific demethylase 1 (LSD1) in the intergenic region of CD103 intron 1, resulting in epigenetic upregulation of CD103 and differentiation of CD103^+^ iTreg cells (73) (Figure 1C). It has been demonstrated that the concentration of TGF-β in the plasma of NSCLC patients positively correlated with the frequency of circulating Treg cells and that TGF-β and Foxp3 were co-expressed in serial sections from tumor tissues of lung cancer (74), implying a contributing role of TGF-β in driving Treg generation in the process of NSCLC development. More importantly, TGF-β signaling blockade has been shown to decrease Treg cell numbers in a lewis lung carcinoma (LLC) mouse model (75), indicating a potential strategy targeting Treg cells for NSCLC treatment. Currently, the TGF-βR1 inhibitor galunisertib is under clinical development in combination with the PD-1 inhibitor, including nivolumab or durvalumab, in NSCLC patients (76).

### IL-10 Signaling

IL-10, an anti-inflammatory cytokine reportedly found at high levels in the cancer microenvironment including in NSCLC (77), is not only an effector, but also a strong inducer of Treg cells (78, 79). IL-10 signals through a receptor complex consisting of two IL-10R1 and two IL-10R2 subunits, which then triggers STAT3 phosphorylation at tyrosine 705 and serine 727 *via* the phosphorylation of the cytoplasmic tails of IL-10R1 and IL-10R2 driven by JAK1 and Tyk2, respectively (80–82). Accumulating evidence indicates that massive production of IL-10 is indispensable for iTreg generation in NSCLC. Hsu and his colleagues (79) found that human monocyte derived-IL-10 potentiated TGF-β-induced iTreg generation, which was abolished in patients with IL-10R gene deficiency and dominant-negative STAT3 mutation. Mechanistic studies revealed that IL-10 not only triggered STAT3 phosphorylation, but also prevented PI3K-Akt activation, both of which induce the transcription factor Foxo1 to translocate into the nucleus *via* a direct protein-protein interaction or phosphorylation (79, 83). Foxo1 then functions as an upstream transcription factor regulating Foxp3 expression during IL-10 stimulated iTreg cell differentiation (84) (Figure 1D).

## Migration of Treg Cells

### CCL22/CCL17-CCR4 Axis

**FIGURE 2:**
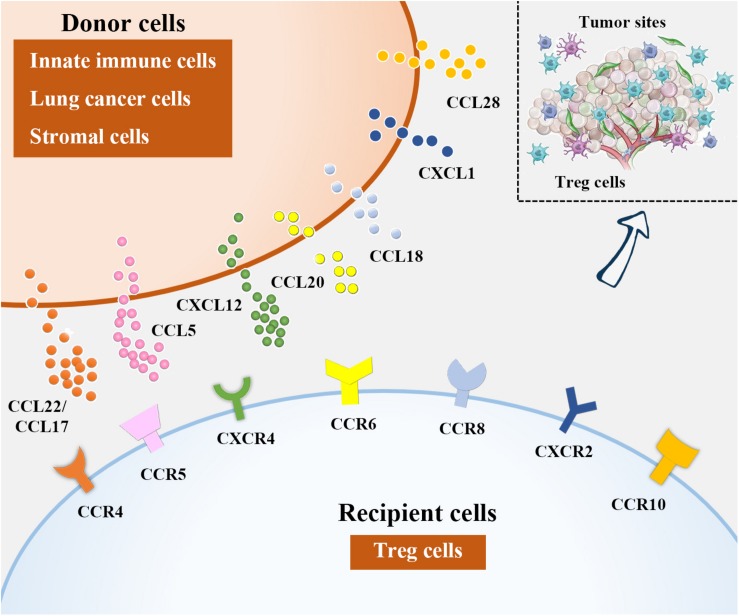
Treg cell recruitment into lung cancer. In NSCLC microenvironment, chemokines attract Tregs by binding to their corresponding receptors. Among them, DCs, macrophages and eosinophils are sources of CCL22, CCL17 is derived from DCs and lung cancer cells, CCL15 can be produced from lung cancer cells and MDCSs, lung cancer cells and stromal cells in NSCLC microenvironment are the major producers of CXCL12 and CCL20, respectively. Besides, CCL18 is generated from DCs and macrophages, lung cancer cells are responsible for CXCL1 and CCL28 production.

Recruitment of Tregs into NSCLC tumor tissues relies on chemokines overproduced by innate immune cells, tumor cells, as well as stromal cells that act through their corresponding receptors (Figure 2). It has been proposed that chemokine (C-C motif) ligand 22 (CCL22), expressed by different immune cells including DCs, macrophages and so on, can induce Treg cell migration by interacting with chemokine receptors such as C-C chemokine receptor type 4 (CCR4) on Treg cells (8, 85, 86). The CCL22-CCR4 axis is currently the most reported mechanism underlying Treg trafficking. TGF-β, the potent inducer of tumor progression and metastasis (87, 88), can augment CCL22 production by reducing the level of its upstream antagonist miR-34a in hepatocellular carcinoma (HCC) (89). Similarly, Wiedemann et al. (90) confirmed that IL-1α-induced Treg migration in HCC was also CCL22 dependent. This precise mechanism may also explain why Treg cells are recruited into NSCLC tumor tissues, as TGF-β and CCL22 are also enriched in these areas (91, 92). Zaynagetdinov et al. (93) found that IL-5, a cytokine involved in allergic and infectious diseases, promotes lung metastasis of tumor cells. Further mechanistic studies revealed that IL-5 contributes to CCL22 production by eosinophils in lung tissues, causing Treg cell recruitment and immunosuppression in the lung cancer milieu.

CCL17, derived from DCs and tumor cells including lung cancer cells, is another reported ligand for CCR4 during Treg cell recruitment (8, 94–96). It has been indicated that the abundance of CCR4^+^ Treg cells attracted by CCL17 was correlated with the clinical stage in NSCLC patients (97). To date, approaches based on Treg depletion in NSCLC patients *via* CCR4 neutralization have shown promise (98, 99).

### CCL5-CCR5 Axis

CCL5 (also called RANTES) is a member of the CC family of chemokines. The roles of CCL5 in cancer biology are versatile as this ligand not only triggers antitumor immune responses but also is implicated in tumor progression and metastasis formation (100, 101). CCL5 has been implicated as a pathogenic factor in NSCLC. By establishing a murine intratibial model of cancer-associated bone resorption, Kim et al. (102) showed that Runt-related transcription factor 3 (RUNX3) silencing in NSCLC cells triggered cell proliferation, migration and invasion, accompanied by osteolytic lesions, by enhancing CCL5 levels, suggesting that CCL5 may be a potent promoter of NSCLC progression. Additionally, Schlecker et al. (103) revealed that MDSC-derived CCL5 infiltration into tumor tissues shared a mechanism similar to CCL5-CCR5 crosslinking to induce Treg cell accumulation.

FOXP3 expression has been reported in several tumors such as melanoma, pancreatic cancer, breast cancer and lung cancer (and is referred to as cancer-FOXP3) despite its non-redundant role in the regulation of Treg cell differentiation, development and functions (104, 105). A positive link between cancer-FOXP3 expression levels and Treg cell accumulation in pancreatic ductal adenocarcinoma (PDAC)-derived tumor tissues was recently shown. Mechanistically, cancer-FOXP3 directly transactivates CCL5, and the CCL5-CCR5 axis promotes Treg cell accumulation in tumor lesions from peripheral blood *in vitro* and *in vivo* (106). Considering that cancer-FOXP3 has been identified as a biomarker for poor prognosis in NSCLC (107, 108), it is rational to propose that FOXP3-initiated CCL5-CCR5 interactions may also participate in Treg cell migration in NSCLC.

The mechanisms underlying CCL5 elevation in the tumor microenvironment are increasingly clear. Nuclear focal adhesion kinase (FAK) was reported to induce Treg cell accumulation in the tumor milieu predominantly by modifying chemokine/cytokine and ligand-receptor crosstalk, including initiation of CCL5 transcription. Mechanistic investigations revealed that FAK integrated with chromatin in the nucleus could form a complex with transcription factors and their upstream regulators that control CCL5 expression (109).

Notably, the migration of Treg cells into tumor areas was reduced when the CCL5-CCR5 signaling was disrupted *via* the reduction of CCL5 production or systemic administration of the CCR5 inhibitor TAK-779 (110), suggesting that selective interventions with CCL5-CCR5 axis might represent a novel immunomodulatory strategy for NSCLC treatment.

### Other Chemokine-Receptor Crosslinks

Reportedly, interactions between CXCL12 and CXCR4 (111), CCL20 and CCR6 (112), CCL18 and CCR8 (113, 114), CXCL1 and CXCR2 (115), and CCL28 and CCR10 (116, 117) are also important contributors to Treg accumulation in NSCLC.

## Heterogeneity of Tregs

Cancer immunotherapies based on Treg blockade have shown sustained clinical responses in NSCLC treatment (118, 119), but their therapeutic efficacy varies and depends partially on the cell phenotype, gene expression, and the functional activities of Treg cells, which endow them with high heterogeneity. In other words, the heterogeneous characteristics of Treg cells make immunotherapy more complicated than ever expected and emphasize a better definition of the cells in lung cancer milieu.

As mentioned above, TCR signaling in response to neoantigens, might determine the heterogeneity of Tregs. By characterizing the TCR profiles of Treg cells from patients with metastatic melanoma, gastrointestinal, and ovarian cancers, Ahmadzadeh et al. (20) showed that the TCR repertoire of intratumoral Treg cells was distinct from that of intratumoral conventional T cells and that tumor antigens can induce the clonal expansion of intratumoral Tregs. Their findings also suggested a dynamic migration of tumor-antigen specific Treg cells from circulation into tumor sites due to the overlap between the TCRB clonotypes of intratumoral Treg cells and those of circulating Treg cells. Thus, it is reasonable that heterogeneous Tregs might receive TCR signaling from tumor antigens to proliferate during lung cancer progression. More importantly, the subtypes and abundances of functional characteristics of activated Treg cells are also heterogeneous in tumor-resident Treg cells, which might be correlated with their immunosuppressive activities.

### Treg-Locking Transcription Factors

By studying the phenotypic, functional, as well as the transcriptional features of Treg cells in 92 NSCLC patients at the single-cell level, Akimova et al. (17) documented that the number and suppressive functions of intratumoral Tregs were dramatically elevated versus those of Tregs in the blood, lungs, and lymph nodes. This group also demonstrated that tumor Tregs exhibited a phenotype of increased abundance of Foxp3, as well as other transcription factors including Eos, IRF-4, Satb1, and Gata-1. Furthermore, the expression of these “Treg-locking” transcription factors was positively correlated with Foxp3 mRNA level, with the highest correlations for Eos and Satb1. Eos had an additional, Foxp3 mRNA-independent, positive correlation with Foxp3 protein level in NSCLC tumor Treg cells (17). Mechanistically, Eos limits Treg plasticity by preventing their reprogramming (120) and IRF-4 and Satb1 promote Treg cell differentiation *via* regulating IL-10 and Foxp3, respectively (121, 122).

### GARP

Glycoprotein A repetitions predominant (GARP), encoded by leucine-rich repeat containing 32 (LRRC32), is expressed on the surface of activated human Treg cells and endows them with TGF-β-dependent bioavailability (123). Exposure to soluble GARP (sGARP) induces Foxp3, represses interleukin IL-2 and IFN-γ production and reduces T cell proliferation, promoting naïve T cells to skew toward iTreg cells. This process is attributed to Smad2/3 phosphorylation (124). Jin et al. (125) found that GARP expression was enhanced in Tregs from the tumor tissues of lung cancer patients and was associated with lymph node metastasis, distant metastasis, and clinical stage, which was partially consistent with Akimova’s findings (17). Furthermore, infiltrating Tregs from patients with early stage lung cancer displayed higher GARP expression than those from patients with advanced cancer, indicating a role for GARP in early diagnosis. *Ex vivo* studies demonstrated that human lung cancer cells might induce the expression of GARP in Tregs by cell contact-independent mechanisms.

### TNFRSF9

To shed light on the baseline landscape of the composition, lineage and functional states of tumor-infiltrating lymphocytes (TILs) in NSCLC, deep single-cell RNA sequencing of 12,346 T cells from 14 treatment-naïve NSCLC patients was performed by Zhang’s group (126). Their findings showed that tumor Treg clonal expansion was mostly cluster-specific and occurred within tumors. Closer examination of the tumor-resident Treg cluster with suppressive functions revealed heterogeneity based on genes correlated with Treg functions. Notably, tumor necrosis factor receptor superfamily member 9 (TNFRSF9), a known activation marker for antigen-specific Tregs, exhibited a striking bimodal expression distribution within tumor-infiltrating Treg cells. Compared to the TNFRSF9^–^ Treg population, the TNFRSF9^+^ population exhibited high levels of a set of 260 genes, including those performing immunosuppressive functions, further supporting the finding that TNFRSF9^+^ cells not only are antigen-experienced but also form the major population of functional tumor Tregs. Survival analysis showed that the 260-gene signature was predictive of worse patient prognosis.

### CD45RA

Treg cells have been proposed to exist in multiple differentiation states with naive, effector and memory phenotypes (127). Two functionally distinct phenotypes of Tregs with suppressive activities, including CD45RA^+^ Foxp3^*lo*^ naive Treg and CD45RA^–^ Foxp3^*hi*^ effector Treg (eTreg) populations, have been identified (128). The tumor milieu is fulfilled with Treg cells with an effector phenotype (129). It has been demonstrated in lung cancer patients that the number of CD45RA^–^ Foxp3^*hi*^ effector Tregs, but not the populations with the naïve CD45RA^+^ Foxp3^*lo*^ phenotype, was increased in TILs compared to the number of those cells in peripheral blood monocyte cells (PBMCs). However, further studies are needed to explore the mechanisms of CD45RA in modulating the suppressive activities of eTreg cells and to evaluate the role of these subpopulations of Tregs in the modulation of NSCLC progression.

## Perspective

Many cancers, including NSCLC, are not easily diagnosed by early laboratorial examination. Immunosuppression is not only a result of cancer progression but also a critical contributing factor for cancer initiation. With the introduction and development of precision medicine together with several developmental sequencing techniques, particularly single-cell sequencing (130), recent advances in examining the mechanisms underlying Treg migration, differentiation and phenotypes have opened new avenues for identifying Treg-based therapeutic strategies for NSCLC. Immune checkpoint inhibitors targeting CTLA-4 and PD-1, as well as neutralizing antibodies against chemokines or the corresponding receptors on Tregs, are showing promise in many cancers such as NSCLC (98, 131). Currently, the burgeoning immunometabolism field has revealed unexpected links to cancer immunology (132, 133). We consider identification of novel mechanisms within this field to be highly valuable, because metabolism disorder may be a mediator between the NSCLC milieu and infiltrating Treg cells. Overall, identification of the precise mechanisms by which Treg cells are recruited, generated or activated in NSCLC tissues not only is essential for improving the understandings of NSCLC pathogenesis but also can provide new insights into other types of cancers with analogous etiologies.

## Author Contributions

MX and JW wrote the manuscript. JX modified this manuscript.

## Conflict of Interest

The authors declare that the research was conducted in the absence of any commercial or financial relationships that could be construed as a potential conflict of interest.
